# The influence of personal and economic oppression on a country's corruption levels worldwide

**DOI:** 10.1016/j.heliyon.2024.e32691

**Published:** 2024-06-13

**Authors:** Yousif Abdelrahim

**Affiliations:** Prince Mohammad Bin Fahd University, Department of Management College of Business Administration, 617, Al Jawharah, Khobar, Dhahran, 34754, Saudi Arabia

**Keywords:** Transparency international human oppression, Institutional oppression, Bribery, Fraud, Illegal enrichment

## Abstract

This empirical research study endeavors to analyze the indirect association between oppression and corruption in 147 nations around the globe to answer one question: "Why does oppression drive corruption in many nations?" The author used secondary data from two different resources. The first source is the Corruption Perception Index (CPI), created by Transparency International (TI) in 2020, and the Human Freedom Index (HFI), co-published by the Cato Institute. In addition, the second source is the Political Stability Index in 2020 to test the three research hypotheses using the R-square, and Anova shows that the model is personal and economic oppression explains 53.5 percent of the variance. In addition, the Weighted Least Squares Regression Analyses imply that there is a positive and meaningful connection between personal oppression (β = 3.028, p < 0.000) and corruption and economic oppression and corruption (5.203, p < 0.000). This study's findings confirmed the theoretical and conceptual relationship between oppression and bribery and identified personal and economic oppression factors as the leading causes of corruption in many countries. The study findings also contribute to the literature and the industry as well. Theoretically, the study results help researchers to understand why oppression causes corruption at the country level. Practically, the study results help policymakers, educators, and international business planners to consider roots when making successful strategic decisions in the era of the globalized world. The author also discussed the research limitations and practical and theoretical implications.

## 1 Introduction

**Fig. 1:**
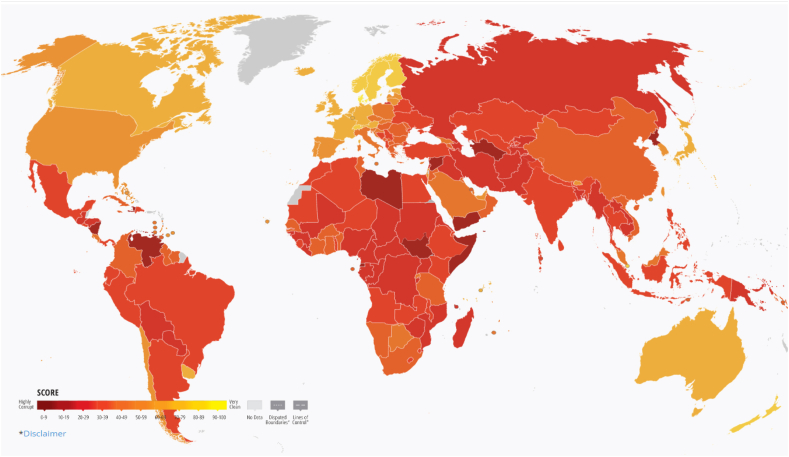
Shows more corruption in countries with less freedom.

**Source:** Corruption Perception Index 2023.

Corruption hurts the needy and helpless the most, raising costs and lessening access to fundamental benefits, such as education, health, justice, and social programs. Corruption aggravates inequality and lessens private sector acquisition to the liability of markets, economies, job opportunities, and economic growth [37]. The literature examination indicates that oppression, which is the opposite of freedom [1], has been related to corruption for a long time [1] (Fig. 1). Fig. 1, borrowed from the Corruption Perception Index 2023, shows how lack of freedom or non-democratic regimes correlate with corruption. Accordingly, North American and Western European countries are less corrupt than the African, South American, Central American, South West Asian, and West Asian countries.

**Fig. 2:**
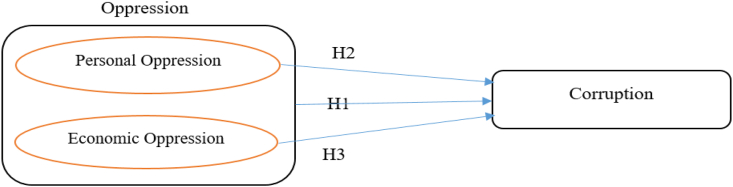
Shows the level of freedom, education, and corruption in Sub-Saharan Africa.

Source: Braddock [42].

Fig. 2 below supports the claim that freedom in Sub-Saharan Africa correlates with the level of corruption among these countries. Additionally, the education theory of Ibn Khaldun [2] backs the claim mentioned above). Ibn Khaldun [2] proposed his theory in education to recommend that school teachers and educators choose and develop teaching methods that fit their students’ stage regarding how to educate future generations and raise ethically educated people by not using punishment and oppression in education. In his theory, Ibn Khaldun believes that students, their teachers, and educators punished for making mistakes or not doing their homework, assignments, or exams are likelier to lie, cheat, or behave unethically to avoid punishment. Hence, oppression minority people who feel oppressed personally or economically are more likely to get away from oppression or get revenge on oppression by behaving unethically via accepting bribes or paying bribes.

## 2 The research gap

Prior studies in the literature suggest that research has yet to empirically examine the relationship between oppression and corruption to confirm or reject that relationship in nations worldwide other than a recent study conducted by Abdelrahim, Eltoum, and Hassan [3]. These researchers have brought other researchers' attention to more precise topics to learn how oppression could influence unethical behavior, such as corruption. However, later researchers should have explained or examined more factors to help examiners understand why or which oppression factors cause corruption. In other words, these researchers have not identified specific oppression factors that cause corruption. Accordingly, the author of this analysis empirically endeavors to scrutinize the connection between oppression and corruption, identify the oppression factors that lead to or cause corruption in many countries, and fill the gap. In other words, this study tries to answer one research question: "What are the oppression factors (economic, religious, personal, political, social, racial, gender, or international trade factors) that influence corruption positively?"

The conclusions of this analysis could aid professors, teachers, and educators in illustrating why intimidation drives corruption in several countries and nations. Likewise, scientific research can have a detailed hypothesis about which corruption aspect causes the majority of corruption in governments and nations. For practitioners, the breakdown outcomes could help teachers, instructors, and government policymakers who create specific training programs that concentrate on battling unethical behavior such as corruption, welcome autonomy schooling, and thus incorruptness. Policymakers must pay more attention to what causes oppression among people in society because oppression not only causes deep mental suffering but also could hurt a country's gross domestic product and human well-being. Hence, these study outcomes might help business managers and supervisors treat their supervisees humanely to maintain employee morale. Finally, the victorious action fighting corruption facilitates prosperity, morals, rightness, and fairness.

## 3 Literature review and hypotheses expansion

### 3.1 Understanding oppression

Based on Cudd [4], oppression is "the absolute unfairness and mistreatment of social institutions and associations in societies." Cudd [4] also considers oppression as "institutional systems performed and conducted on clusters by different groups using explicit and avoiding material and inspirational powers that violate fairness." Additionally, Cudd [4] argues that avoiding material powers. For example, roughness and financial poverty drive social intimidation. Such characteristics impact an individual's ethical personality in a forced and impaired way.

Hence, being vulnerable to the previously cited manners and procedures has the promise to harm the personality of tyrannized individuals [5,6]. It is essential to express that corruption and oppression could be brought into existence and promoted by communities. Rousseau [7] claims that oppression can only arise when victimized individuals are inclined to surrender their liberty for some compensation that is obvious only to people in a civilized society. People who suffer from oppression in society are usually treated ruthlessly or are deprived or denied from having the same freedom, opportunities, help, rights, and benefits as others. Oppressive behavior differs from person to person. Three tiers of oppression—internalized, institutional, and interpersonal—are connected, and all three classes of oppression spread and strengthen one another [8]. Institutional oppression is the recurring injustice of individuals within a social identity class, reinforced and executed by the community and its organizations. Institutional oppression is exclusively based on the individual's membership in the social identity class [9]. Internalized oppression occurs when associates of an oppressed class tolerate or reaffirm harmful stereotypes averse to their group [10]. Interpersonal oppression is the belief that one class is better than other classes and has the privilege to oversee or control the other. Institutions shape interpersonal oppression into society and grant approval and support for the controlling group members to personally mistreat or disrespect individuals in the oppressed class [9]. Hence, a state of oppression will be maintained when all three tiers of oppression work together. Oppressive behavior can take multiple forms, varying from hostile comments constructed in ignorance to physical violence, insults, and threats [8].

### 3.2 The concept corruption

TI [11] defines corruption as the regime, Administration, non-government management, administrators, and employees abusing authority with decision-making roles for personal interest and gains. The behavior of corruption comprises bribery, illegal enrichment, manipulating national aid, employing fellows and connections, and stealing. Caiden [12] claims several sources incorporate corruption, including psychological, economic, ideological, political, external, technological, and socio-cultural and technological factors. Research investigators such as Seldadyo and De Haan [13] specified financial and non-economic determinants and cultural aspects that construct corruption among nations. Rose-Ackerman [14], furthermore, always describes corruption as the "misapplied of shared authority for personal or governmental earnings." Rose-Ackeman [14] has provided illustrations of corrupt actions, including deception, spending and accepting illegal payments, conflict of interest, and theft. Furthermore, Uslaner [15] asserts that communities assemble "civilizations of corruption" since "a cruel rotation of increased imbalance, inferior out-group faith, and elevated corruption trap communities." Likewise, Uslaner [15] claims that individuals in unethical civilizations share in unethical circumstances not for enjoying their sinful heads but for being forced, and they have no other options.

### 3.3 The relationship between oppression and corruption

Several researchers have examined and debated the relationship between oppression and corrupt practices theoretically and empirically. For example, Prasad et al. [16] directed the ethnographic literature on corruption. Prasad et al. [16] also argue that house cleaners and familiar people commit corrupt conventions in admission to restrain from racial and lineage clusters or as a manner to handle preceding oppression. Khaldun [17], who declared that oppression guides corruption, also discussed that oppression affects moral nature. Furthermore, the theory of education developed by Ibn Khaldun exemplifies the connection between intimidation and corruption. The core of Ibn Khaldun's theory is that tyrannized individuals will be unethical because intimidation affects a person's manners to understand how to deceive, defraud, and embrace all types of unethical conduct [2]. The sources of corruption lie in lawful imbalance, economics, inadequate approach options, and an inferior level of widespread faith, backed by this opinion [15]. In addition, Amundsen [18] revealed a contrary association between democratic systems and political lawlessness.

Furthermore, oppression, contrary to liberation, has been connected to corruption for a long time [1]. Moreover, McLaughlin [19] has examined power distance (PD) as a type of corruption. As declared by Hofstede and Hofstede [20], PD denotes the extent to which small influential associates of organizations and associations anticipate and bear that society does not unjustifiably distribute power. Unequally allocated power points oppression and absence of independence, McLaughlin [19] inferred that corruption also increases as PD within a nation rises. Hence, the author argues that oppression is one of the factors that positively influences corruption and posits hypothesis one (H1):

H1 High-oppressed nations are more likely to be more corrupt than low-oppressed nations because oppression deteriorates people's values of integrity, truthfulness, sacredness, and spiritual faith.

### 3.4 Economic oppression and personal oppression versus oppression

According to Cudd [4], economic oppression is paramount to all kinds of oppression. Hence, researchers need to pay more attention to investigating economic corruption. Multiple reasons initiate economic oppression, which hurts people's well-being, economic equality, and lives. These reasons include structural brutality, military blockades, and human privilege violations [21]. The literature review shows that financial oppression might take many forms, including, though not restricted to, low wages, coerced labor, deprivation of similar chance, practicing career discrimination, bonded labor, and financial prejudice founded on race, sex, religion, and ethnicity [22]. This qualitative study is critical to understanding and conceptualizing the many forms of economic oppression. Ann Cudd defines the significant powers of economic oppression as straightforward and indirect influences. Although socialism and capitalism are not intrinsically dictatorial, they "loan themselves to intimidation in typical forms" [4]. Dobel [23] claims that inequality in countries and societies with unequal economic and authority distributions has generated forces that have alienated individuals and guided them to social breakdowns. Social breakdown drives moral changes among individuals in a society; therefore, some individuals lose ties to their excellent moral principles because of the anomic conditions and behave unethically to achieve personal goals [24]. In addition, the theory of corruption, which brings together the economic, moral, social, governmental, and political motivations and ways of bribery in one hypothetical framework [23], implies that people's immorality created by moral changes due to economic inequality is a cause of corruption. Hence, people who feel oppressed due to their deprivation from decent jobs, social status, or political positions are more likely to behave unethically and be involved in corrupt behaviors. Following the discussion mentioned above, the author posits hypothesis two (H2) and hypothesis three (H3):

H2 High-oppressed individuals are more likely to be corrupt than low-oppressed individuals because oppression deteriorates people's values of integrity, truthfulness, and sacred and spiritual faiths.

H3 High-oppressed economies are more likely to be corrupt than low-oppressed economies because oppression deteriorates people's values of integrity, truthfulness, and sacred and spiritual faiths.

## 4 Research methods

### 4.1 Collection of data

The research writer employed the subsequent unrestricted research representative to assemble secondary data from various data resources. In addition, the author examined the examination analysis hypothesis below:

(1) KOG=β0+β1*OPP+€

Where KOG, OPP, and € denotes corruption, oppression, and error, respectively.

(2) KOG=β0+β1*PerOP+€

Where KOG, PerOP, and € denotes corruption, personal oppression, and error, respectively.

(3) KOG=β0+β1*EcoOP+€

KOG, EcoOP, and € denote corruption, Economic oppression, and error, respectively. Following the examination of the above hypothesis breakdown, the examination-specific representative that mirrors the WLSRA examination downward shows the association between personal (oppression) intimidation and corruption, as well as economic oppression, are positive and significant:

(4) KOG=β0+β1*PerOP+€

(5) KOG=β0+β1*EcoOP+€

### 4.2 The dependent variable

The dependent variable is corruption. The author estimates at the national level. The author used the CPI as a rep to calculate corruption in 133 territories and countries. TI constructed the CPI: specialists and researchers rate 179 territories, regions, and nations established on assessments of corruption. These researchers and specialists rate corruption using survey queries from the previous twenty-four months in twelve diverse organizations. The CPI incorporates datasets from thirteen distinct references investigating a nation's trade individuals and specialists' thoughts on the state sector's corruption. The CPI put together multiple measurements of corruption to comprise an unrestricted online index at https://www.transparency.org/en/cpi/2022. Survey respondents who are professionals and enterprise administrators replied to the CPI questionnaire that identifies every type of corruption in the state departments, including patronage, favoritism, nepotism, and bribery. The CPI utilizes a span of zero (extremely corrupt) to one hundred (very low corrupt). Denmark, for instance, represents the highest with a score of 88, whereas countries such as Somalia State, with nine, stand at 179.

### 4.3 The independent variable

Personal and economic oppression are the independent variables of this study, gauged at the national level among one hundred and fifty-three nations around the Globe by the Human Freedom Index (HFI). The HFI displays the essence of human liberation in the Globe. The base of the HFI is the overall estimate that comprises civil, personal, and economic freedom. The HFI recognizes human autonomy as a cultural notion with the value of people and as unfavorable freedom or the nonexistence of coercive constraint. The HFI is a comprehensive freedom index created for a globally meaningful group of nations and countries. The HFI incorporated one hundred sixty-two nations and governments in 2022, the recent and current year for which sufficient dataset is feasible. The HFI measure varies from zero (0) to ten (10), where the number ten (10) denotes more additional freedom in a nation, whereas the number zero denotes not-so-autonomy in a country. The HFI essay is co-published by the Cato Institute and the Fraser Institute. The HFI assembled their dataset free at https://www.cato.org/human-freedom-index/2020.

### 4.4 Control variables

The publications in the literature review examination show that a nation's governmental volatility can influence the country's level of corruption. State flux has boosted corruption in the long term [25]. The political instability index assesses governmental instability. Researchers can get free access to the Global Economy via the Economist website at https://www.theglobaleconomy. com/rankings/wb_political_stability/. The Economist Study arranges all countries at a benchmark running from zero (0) to number ten (10), where the number zero (0) suggests a nation is not corrupt, and the number ten (10) denotes a country is highly corrupt.

### 4.5 Hypotheses test

In this research analysis, the author analyzed the influence of personal and economic oppression on corruption in one hundred and fifty-three nations employing the weighted least squares regression analysis. In addition, the author controlled political instability since political instability could influence both oppression (personal and economic) and corruption. The author uses the nations' political instability as a control variable to examine the effect of individual and economic oppression on corruption levels among nations and obtain more accurate outcomes. As said above, a country's political instability in nations can affect the nation's corruption level. The author utilized the WLSRA for experimenting with the simple regression examination assumptions indicating the abnormal distribution of the corruption dataset since the Shapiro-Wilk examination is significant (β < 0.05). Unlike the linear and nonlinear least squares regression approach, WLSRA does not connect with a specific type of operation employed to demonstrate the link between the function variables. Instead, weighted least squares display the interpretation of the model's spontaneous errors; these errors apply to linear or nonlinear operations in the parameters. The errors function by incorporating more nonnegative weights, or constants, corresponding to each data pinpoint, into the appropriate model. The weight proportions denote the data's precision incorporated by the examiner in the related observation. Optimizing the suitably weighted standard to determine the parameter patterns permits the weights to specify the contribution of every observation to the prior parameter estimations [26].

Nevertheless, the weighted least-squares regression approach, like the other least-squares techniques, is likewise suitable for the effects of outliers. If outliers are not studied and dealt with adequately, they will negatively affect the parameter estimate and other elements of a weighted least-squares estimation. Based on Modern Regression Methods, if a weighted least-squares regression raises the effect of an outlier, the examination results might be far less than an unweighted least-squares examination [27].

### 4.6 Findings

All through the outcomes of the Weighted Least Squares Regression Analyses technique (Table 1), the researcher of this examination endeavors to explain the deviations worldwide in the tendency to be corrupt. Table 1 depicts how all examined variables correlate in the Weighted Least Squares Regression Analyses. Table 2a, Table 2b Model 1 and Table 2a, Table 2b Model 2 indicate the propensity of a country to be corrupt versus personal oppression, economic corruption, and the country's political stability. The all-nations cross-sectional dataset for corruption under examination assesses corruption perceptions in one hundred fifty-three countries. The outcomes from the WLSRA examination imply that personal oppression (Per-Oppression) and economic oppression (Eco-Oppression) are two of the significant factors. These two factors impact the level of corruption in several countries since both are meaningful significantly in the WLSRA equation incorporated in this analysis (β1Per-Opression = 0.2356884, the p-value is less than 0.000; β2 Eco-Oppression = 0.1238071, the p-value is less than 0.000). Personal and economic oppression seem to have a notable and positive connection with the nation's corruption. Eventually, a country's political stability appears to be an influential variable to control for in the regression equations (β1 = 11.827, p-value<0.000).

**Table 1:** Results from Regression analysis using the Weighted Least Squares Regression Analyses Regarding Statistics and Correlations.

	Corruption	Political Instability	PerOpp.	EcoOpp.
Corruption	1			
Political Instability	0.767**	1		
PerOpp.	0.723**	0.746**	1	
EcoOpp.	0.696**	0.647**	0.631**	1
*Mean*	44.67	−1.02788	−2.9987	−2.8898
*STD*	18.745	0.914033	1.4444	1.05586

**Note:** (N = 147): the **Correlation is significant at 0.01 level (2-tailed); STD= Standard Deviation; PerOpp. = Personal Oppression; EcoOpp. = Economic Oppression; PI= Political Instability.

**Table 2a:** **Model 1** The Weighted Least Squares Regression Analysis results show beta values and the significance of the T-change for a country's political instability versus personal and economic oppression.

	β (Beta value)	t-Change l Significance
Constant	47.032	47.795***
Political Instability	15.982	14.791***
R - Square	0.588	218.759***

**Note:** (N = 147): **Correlation is significant at 0.01 level (2-tailed); Opp. = Oppression; Cor. = Corruption.

**Table 2b:** **Model 2:** The Results from the Weighted Least Square Analysis show the impact of personal and economic oppression on corruption after controlling for political instability Regarding Beta Value Significance.

Variables	β (Beta value)/Significance
Constant	53.72270.051***
PerOpp.	3.028**
EcoOpp.	5.203**
Political Instability	11.827***
R - Square	0.595***

**Note:** (147): ***Correlation is significant at 0.001 level (2-tailed); **Correlation is significant at 0.01 level (2-tailed) *Correlation is significant at 0.05 level (2-tailed).

## 5 Conclusions

### 5.1 Theoretical and practical implications

The author of this study hypothesizes that high-oppressed nations are more likely to be corrupt than low-oppressed nations because oppression deteriorates people's values of integrity, truthfulness, and sacred and spiritual faith. In addition, the author also hypothesizes that High-oppressed individuals are more likely to be corrupt than low-oppressed individuals because oppression deteriorates people's values of integrity, truthfulness, and sacred and spiritual faiths. The WLSRA study reinforced this analysis of Hypothesis One (H1) and Hypothesis 2 (H2). In other words, empirical oppression has a powerful effect on the nation's corruption level.

The study outcomes affirm the meaningful connection between the oppression levels in a country and corruption among nations and governments and, with the empirical examination, added confirmation of facts to the publications. H1 is compatible with the findings of Abdelrahim, Eltoum, and Hassan [3], who examined the relationship between oppression as a dimension in general and corruption in countries without the two other elements that constitute oppression to determine precisely which oppression factor causes corruption. In addition, these study results also align with Partow, Moridnejad, Irannezhad, Parizadeh, and Dadpour [39], who confirm that economic oppression practiced by the Iranian regime in the education sector causes corruption. Furthermore, the study results align with Trianingtyas and Anam's [40] findings. These researchers found that oppression happens because the capitalist class wishes to retain its control and status quo. Moreover, this study's findings also support Hafez, Abd El Aziz, and Mahmoud's findings that argued that African-Americans and Nigerians have encountered oppression, violence, racism, marginalization, social inequality, and discrimination caused the Europeans have suffered from corruption [41].

The Human Freedom Index, a comprehensive measure of human freedom, defines oppression as a combination of economic, personal, and civil elements that restrict human freedom and foster oppression. This index, along with Baddock's [1] theoretical link between oppression and corruption, forms the basis of our discussion. The study results, which confirm that oppression leads to corruption in certain countries, underscore the importance of personal and economic factors. These findings are in line with Ibn Khaldun's theory of education [2] and Uslaner's [15] proposition that corruption stems from inadequate policy alternatives, economic factors, a lack of widespread faith, and legal imbalance.

Likewise, Amundsen [18] proved that there is an opposite affinity between political corruption and democracy. This study's results make sense since oppression deteriorates people's values of integrity and spiritual faith and affects their moral nature. The Institutional Anomie Theory developed by Messner and Rosenfeld [28] postulates that losing some cultural norms and values disconnects individuals from their social norms and laws and creates anomic conditions that make people behave unethically and justify their unethical behavior [29]. Likewise, in hypothesis three (H3), "High-oppressed economies are more likely to be corrupt than low-oppressed economies because oppression deteriorates people's values of integrity, truthfulness, and sacred and spiritual faiths." Oppressed people are more likely to be corrupt once their moral integrity deteriorates, anomic conditions are created, the disconnection from their social norms and rules will at hand, and people can find themselves behaving unethically and practicing corruption in different ways with their justifications.

Moreover, researchers such as Braddock [1] have acknowledged that oppression is associated with corruption and more elevated corruption levels in many nations. Theoretically, this analysis study's findings and conclusions could help educators describe why oppression drives corruption in some countries. Also, investigators could have a detailed conception concerning which corruption aspect delivers the majority corruption levels in societies worldwide. Practically, the outcomes of the analysis could help professors who tailor specific practicum school curricula that concentrate on dishonest behavior, such as corruption, to think about adopting autonomy, liberty, and honesty.

**Fig. 3:**
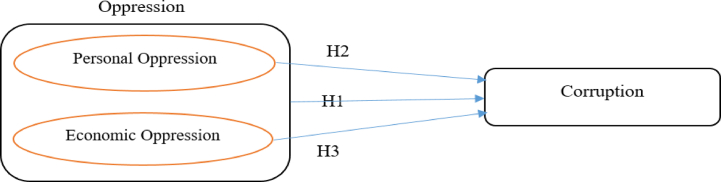
Shows the conceptual framework for the relationship between oppression, personal oppression, economic oppression, and corruption.

Source: The Author Work.

This study's empirical findings of the three supported hypotheses study align with the theoretical and conceptual findings of [1], Prasad et al. [16], Uslaner [15], and Ibn Khaldun's theory of education. All those scholars have theoretically linked oppression to corruption and ethical behavior. In addition, this study's findings support the empirical findings of Abdelrahim, Eltoum, and Hassan [3]. Unlike prior study findings, this study confirmed the theoretical and conceptual relationship between oppression and corruption and identified the exact factors that cause corruption (i.e., personal oppression and economic oppression)(Fig. 3) In his theory, Ibn Khaldun believes that students, their teachers, and educators punishing them for making mistakes or not doing their homework, assignments, and exams are likelier to lie, cheat, or behave unethically to avoid punishment. Hence, oppression minority people who feel oppressed personally or economically are more likely to get away from oppression or get revenge for oppression by behaving unethically via accepting bribes or paying bribes, which is a form of corruption. These researchers have brought other researchers' attention to more precise topics to learn how oppression could influence unethical behavior, such as corruption.

School administrators could include policies in their school system to guide their teachers in not oppressing students personally via any physical or emotional stress if they want to raise honest future employees who are not corrupt. These future employees are university and college. Hence, students' well-being (i.e., being happy, healthy, and secure) is essential to school educators, who must be oppression-free. It is critical for policymakers to pay more attention to what causes oppression among people in society [35] because oppression not only causes deep mental suffering but also could hurt a country's gross domestic product and human well-being. Policymakers, college, university, and school administrators can use this study's findings by incorporating professional development training sessions for teachers and professors to help them understand the importance of students not being oppressed [30] and how they can provide effective learning with more freedom and less oppression. Hence, these study outcomes might help business managers and supervisors treat their supervisees humanely to maintain employee morale.

Prospective research should investigate the impact of oppression on the corruption levels of individuals who might feel enslaved [36] and firm levels to comprehend the origin of oppression at different levels. Moreover, the conclusions of the analysis also provide directions for policymakers to raise awareness of oppression regarding the socioeconomic population. Ultimately, the study outcomes might help managers in business firms adopt strategies to crush legal, societal, political, artistic, personal, and institutional oppression.

Oppression in society primarily arises from the systems of anti-Semitism, ageism, sexism, racism, heterosexism, or unfairness. For example, restricting access to people with no justification, creating unequal housing or institutions for people by status or connections, and diverting financial resources from low-income citizens are some forms of economic oppression. Policymakers who are eager to fight corruption and unethical behaviors to benefit from these study findings to come up with anti-oppression strategies that actively challenge oppression on an ongoing basis in one's daily life and foster social justice and, therefore, oppression. In addition, the study findings can help lawmakers and legislators to pass and apply strict laws that tackle all forms of oppression for those who practice oppression. In other words, personal and economic oppression should be criminalized for its severe consequences in society as a whole economically. Psychologically, oppression-based trauma and stress and trauma also arise when oppressed people experience fear and worry, and can lead to poor mental health that negatively impacts society through unhealthy working people with low or no productivity. This study's findings could help psychologists and educators support their argument and develop education programs and training courses to help reduce the number of victims of oppression. For example, using this study's findings, policymakers could write policies, rules, and regulations based on the following:

1. reveal corrupt exercises and threats via transparency.
2. maintain the general sector honest, accountable, and transparent.
3. prevent unfair and illegal practices.
4. Confirm that public sector workers function in the public interest.
5. Support educators and psychologists in developing and applying educational programs that prevent oppression, unethical behavior, and corruption.

### 5.2 The study limitations

The study's research limitation can be summarized as follows: Firstly, this study analysis focuses on the impact of personal and economic oppression within a nation on the country's corruption level, not the individual or firm level, using secondary data from three different resources to answer specific research questions. The prospective examination could analyze which type of oppression causes the majority of influence on individuals' corruption; is it societal oppression? Is it political oppression? Alternatively, is it workplace oppression? To answer these questions, researchers need to investigate economic and personal oppression deep in several areas, including the rule of law, religion, relationships, security and safety, sound money, movement, freedom of information and expression, size of government, freedom to international trade, regulations, legal system and property rights. This study examined personal and economic oppression because the evidence in the literature review supports the arguments discussed throughout the paper. The author used secondary data from two different resources. The first source is the Corruption Perception Index (CPI), created by Transparency International (TI) in 2020, and the Human Freedom Index (HFI), co-published by the Cato Institute. In addition, the second source is the Political Stability Index in 2020 to test the three research hypotheses using the R-square. ANOVA shows that the model fits the variance, which indicates that personal and economic oppression constitutes 53 % of the variance explained. Hence, this study is limited to secondary data. Future studies can use primary data to confirm or refute the relationship between oppression and corruption. It is not always true to generalize findings at the country level as if they were true on individuals or firms. Furthermore, this study controlled for one factor that influences oppression and oppression. However, future research may add more control factors should they find support from the literature.

Moreover, future research should include civil oppression since oppression encompasses civil, economic, and personal oppression. For example, oppression reduces freedom, jeopardizing human dignity and honor [38]. In addition, future research should also examine the relationship between personal oppression, economic oppression, civil oppression, cultural oppression [31], and corruption using primary data. Researchers who employ primary data acquire more in-depth insights from respondents. Surveys may not cover some respondents' opinions and experiences.

Moreover, this study controls for one political factor (i.e., political instability) influencing the dependent and independent variables. However, there might be more variables to control if there is evidence in the literature and the data available to support the claim and justify the control for any additional control variables. This study is limited to high-income and low-income countries. Some researchers find that low-income countries are more corrupt than high-income countries [32]. However, Huberts [33] claims that "whether this association is causal is unclear. Hence, future research should examine the relationship between oppression, economic oppression, personal oppression, and corruption in low-income and tribal countries with high levels of personal oppression and corruption [34]. Only then will the examination be repeated in high-income countries to confirm or refute the Graaf [32] claims. Finally, this study is limited to one control variable (i.e., political instability) for data availability and the supporting evidence in the literature. Future research could control for other socio-economic factors should they find evidence and data.

## Funding

The author of this manuscript received no monetary aid for the study, authorship, and journal publication of this paper. He reports a relationship with his current university, which includes employment.

## Data availability statement

The author used secondary sets, available online to researchers free and without restriction (Table 3). Has the data associated with this study been deposited into a publicly available repository? The answer is no. My study provided online links to publicly accessible data.

**Table 3:** The secondary data sources and the online links to the sources.

Variable Name	Data Source	Data Online Link
Corruption	Corruption Perception Index	https://www.transparency.org/en/cpi/2022.
Personal Oppression	The Human Freedom Index	https://www.cato.org/human-freedom-index/2020
Economic oppression		
Political Instability	the political instability index	https://www.theglobaleconomy.com/rankings/wbpolitical_stability/

## CRediT authorship contribution statement

**Yousif Abdelrahim:** Writing – original draft, Supervision, Software, Project administration, Methodology, Formal analysis, Conceptualization.

## Declaration of competing interest

The authors declare the following financial interests/personal relationships which may be considered as potential competing interests:Yousif Abdelrahim reports a relationship with 10.13039/501100013781Prince Mohammad Bin Fahd University that includes: employment. If there are other authors, they declare that they have no known competing financial interests or personal relationships that could have appeared to influence the work reported in this paper.
